# Barriers to and facilitators for implementing quality improvements in palliative care – results from a qualitative interview study in Norway

**DOI:** 10.1186/s12904-016-0132-5

**Published:** 2016-07-15

**Authors:** Ragni Sommerbakk, Dagny Faksvåg Haugen, Aksel Tjora, Stein Kaasa, Marianne Jensen Hjermstad

**Affiliations:** European Palliative Care Research Centre (PRC), Department of Cancer Research and Molecular Medicine, Faculty of Medicine, NTNU, Norwegian University of Science and Technology and St. Olavs Hospital, Trondheim University Hospital, P.O. Box 8905, N-7491 Trondheim, Norway; Cancer Clinic, St. Olavs Hospital, Trondheim University Hospital, Trondheim, Norway; Regional Centre of Excellence for Palliative Care, Western Norway, Haukeland University Hospital, Bergen, Norway; Department of Sociology and Political Science, Norwegian University of Science and Technology, Trondheim, Norway; Department of Oncology, Oslo University Hospital and University of Oslo, Oslo, Norway; Regional Centre for Excellence in Palliative Care, Department of Oncology, Oslo University Hospital, Ullevål, Oslo, Norway

**Keywords:** Palliative care, End-of-life, Barrier, Facilitator, Quality improvement, Implementation strategy, Organization of care, Cancer, Dementia, Norway

## Abstract

**Background:**

Implementation of quality improvements in palliative care (PC) is challenging, and detailed knowledge about factors that may facilitate or hinder implementation is essential for success. One part of the EU-funded IMPACT project (IMplementation of quality indicators in PAlliative Care sTudy) aiming to increase the knowledge base, was to conduct national studies in PC services.

This study aims to identify factors perceived as barriers or facilitators for improving PC in cancer and dementia settings in Norway.

**Methods:**

Individual, dual-participant and focus group interviews were conducted with 20 employees working in different health care services in Norway: two hospitals, one nursing home, and two local medical centers. Thematic analysis with a combined inductive and theoretical approach was applied.

**Results:**

Barriers and facilitators were connected to (1) the innovation (e.g. credibility, advantage, accessibility, attractiveness); (2) the individual professional (e.g. motivation, PC expertise, confidence); (3) the patient (e.g. compliance); (4) the social context (e.g. leadership, culture of change, face-to-face contact); (5) the organizational context (e.g. resources, structures/facilities, expertise); (6) the political and economic context (e.g. policy, legislation, financial arrangements) and (7) the implementation strategy (e.g. educational, meetings, reminders). Four barriers that were particular to PC were identified: the poor general condition of patients in need of PC, symptom assessment tools that were not validated in all patient groups, lack of PC expertise and changes perceived to be at odds with staff’s philosophy of care.

**Conclusion:**

When planning an improvement project in PC, services should pay particular attention to factors associated with their chosen implementation strategy. Leaders should also involve staff early in the improvement process, ensure that they have the necessary training in PC and that the change is consistent with the staff’s philosophy of care. An important consideration when implementing a symptom assessment tool is whether or not the tool has been validated for the relevant patient group, and to what degree patients need to be involved when using the tool.

**Electronic supplementary material:**

The online version of this article (doi:10.1186/s12904-016-0132-5) contains supplementary material, which is available to authorized users.

## Background

Palliative care (PC) aims to relieve suffering and preserve the best possible quality of life until death for patients whose disease is not responsive to curative treatment [1]. Important components of PC are the interdisciplinary approach, the control of pain and other symptoms and that the physical, psychological, social and spiritual needs of patients and their families are met [2].

Traditionally, PC services have focused on patients with advanced-stage cancer [3]. Due to the aging of the European population, the cancer incidence is expected to increase dramatically, with more than a 20 % increase from 2002 to 2020 [4]. This will also lead to a higher prevalence of cancer as more patients live longer with metastatic disease, often resulting in a complex symptom situation. An aging population also means an increasing number of people with other progressive, life-limiting chronic diseases [3, 5], dementia in particular [6]. Thus, in the years to come, there will be an increased need for PC, in a variety of health care settings [7].

To meet the increased demand, efficient strategies are necessary to implement and/or improve PC at all levels of health care, not just in specialist settings. However, reviews show that while certain implementation strategies (such as educational training, reminders, audit and feedback) might be effective in some settings, they might not work in others [8]. When planning an improvement project, an important early step is therefore to identify potential barriers and facilitators for implementation of changes in that particular setting [9]. One aim of the EU-funded project IMPACT (IMplementation of quality indicators in PAlliative Care sTudy) has been to identify these influential factors. During three phases of the IMPACT project, researchers in England, Germany, Italy, Norway and the Netherlands have explored factors influencing the implementation of improvements in services providing PC for patients with cancer and/or dementia [10]. In the first phase, common challenges to providing good quality PC in the five countries were explored through interviews with policy makers, researchers and health care providers [11]. In the second phase, an exploratory study using retrospective interviews with health care providers identified common factors that had influenced previous improvement projects in PC in the five countries [12]. In the final phase, barriers and facilitators were identified through a process evaluation of improvement projects in 40 services during a pretest-intervention-posttest study.

A publication summarizing the results from the phase two interviews in all five countries has been published elsewhere [12]. The present paper presents detailed findings from the Norwegian interviews.

### Palliative care in Norway

Norway has a public health care system, with hospital services funded by the central government and community services (nursing homes and home care) funded by the local government. PC services have developed over the past two decades. There are essentially no private or charity PC providers. The Directorate of Health has issued national norms and standards for PC at the different health care levels [13]. Basic PC is provided in hospital wards, nursing homes, and municipal home care services as an integrated part of general patient care. Specialist PC is provided by hospital-based, ambulatory PC teams serving hospital wards and community services, in designated hospital inpatient units, and in designated units or beds in nursing homes. There is good national coverage of specialist PC teams and hospital units as well as PC or oncology nurse specialists in the communities, while nursing home units are more unevenly distributed [14]. The introduction of the Coordination Reform in 2012 has had implications for PC. One objective of this national health system reform is to ensure that the municipalities take on more responsibility for the care of patients. The patients are therefore discharged from specialist services earlier than before.

Specialist PC has predominantly been offered to cancer patients, but the proportion of non-cancer patients is increasing, especially in community services. The development of PC services is actively supported by the central government and well accepted by the general public. However, there is still insufficient integration between palliative medicine and other medical specialties, and a need for PC expertise in dementia care [14].

Few studies have been published on factors influencing the implementation of improvements in PC in the Norwegian context, and those identified concern the introduction of computer-based symptom assessment. Both André et al. [15] and Fyllingen et al. [16] found that the patients’ physical and cognitive status had an effect. André et al. also found that certain aspects of the work culture [17] and “conflicts between ‘high tech’ and ‘high touch’” [15] were barriers to implementation, while having an in-house resource person who could provide help and training was a key facilitator [18].

### Aim

This article aims to answer the following research questions:

1. Which barriers to and facilitators for implementing improvements in PC have been experienced by health care providers in Norway?
2. Which of the experienced factors can be perceived as being particular to PC?

## Methods

A qualitative research approach using semi-structured interviews was applied. This method was chosen because of the flexibility it offers in exploring individuals’ experiences [19]. The present article includes the findings from seven individual interviews, two dual-participant interviews and two focus group interviews conducted with managers and health care providers in Norway between April and June 2012.

### Sampling and data collection

The participating services were all recruited from the same region in Central Norway and were purposely selected. The intention was to recruit the same number and types of services that would be included in a subsequent IMPACT intervention study. However, we did not succeed in recruiting any home care services in time for this interview study, due to difficulties getting access to the services. Thus, the Norwegian services consisted of two hospitals, one nursing home and two local medical centers. A local medical center (LMC) is a primary care service that offers short term in-patient care, and has higher staffing levels of physicians, nurses and physiotherapists than nursing homes. One of the centers had a PC in-patient unit (LMC-PCU), while the other specialized in geriatrics and dementia care (LMC-GU). The nursing home was an ordinary nursing home with one of the wards specializing in dementia care. Three hospital services participated in the study: a specialist PC unit (H-PCU) and a geriatric unit (H-GU) in a regional university hospital, and a PC consult team (PCT) at a local hospital.

The researcher contacted the executive director or the head physician of each service by e-mail describing the study and asking if their service was willing to participate. Professionals from these services were included if they were directly involved in providing clinical PC (nurses and physicians) or involved in the organization of PC (managers)*.* They also had to have at least one year working experience within the service. All together one male and 19 females were included in the interviews. They were between 25 and 65 years old (mean age: 51) and had between 1.5 and 20 years of experience providing PC. The head nurses in the dual-participant interviews and the participants in the individual interviews were all contacted directly by the researcher, while the participants in the focus groups were chosen by the head nurses of the two PC units.

Individual interviews were conducted with two executive directors, one head nurse, three physicians and one quality improvement nurse (QI nurse). A QI nurse has particular responsibility for quality improvement projects and for training staff and students in the given service. The two dual-participant interviews were not planned as such. However, the head nurses in these two services also wanted the QI nurse or the assistant head nurse to participate. All but one of the interviews were conducted at the interviewees’ workplace during working hours, the last one by phone.

Each of the two focus groups with staff included 4–5 participants working in specialist PC in-patient services. To include as many staff members as possible, the focus groups were conducted between shifts. All the participants were nurses. We had asked for both female and male nurses for the focus groups, but only one male was able to participate on the scheduled days. The first author (RS), a sociologist with experience in conducting qualitative interviews, conducted the seven individual interviews and the two dual-participant interviews alone, while the two focus groups were managed through collaboration between RS and a research assistant. The research assistant asked most of the questions, while RS ensured that all topics were covered and followed up with additional questions along the way. The interviews took between 24 and 112 min with an average length of 62 min.

All interviews were conducted using a semi-structured interview guide (Additional file 1). The interview guide was developed by the Dutch IMPACT research team and consisted of the following three themes: 1) known improvement strategies; 2) barriers and facilitators; and 3) potential strategies. Questions were refined during an international meeting with researchers from the IMPACT project, and translated into Norwegian by the authors. The interviews were audio-recorded and transcribed verbatim.

### Analysis

The data were analyzed in two separate rounds, both guided by thematic analysis [20]. During the first round, an *inductive coding approach* [20] was used and the suggested codes were closely related to the text fragments. To control for inter-coder variability, two researchers read and coded the interview transcripts separately, compared results and agreed on the name and definition for each code. After two interviews had been conducted, codes and associated text fragments were translated into English and shared between researchers during an international IMPACT meeting to develop a cross-national consensus codebook for the larger interview study. This codebook was used to assist the researchers in the analysis of the remaining interview and focus group data. However, new codes were continuously developed during the analysis process. To ensure that all relevant text fragments had been identified, all transcripts were reread as new codes were developed. ATLAS.ti computer software [21] was used to sort the text fragments according to code. Themes were derived from the codes and discussed between the researchers in the five countries via e-mail and Skype. A report was produced in each country, summarizing the findings on barriers to and facilitators for improving the organization of PC.

During the second round of data analysis, a *theoretical thematic approach* [20] was applied to the Norwegian transcripts using Grol and Wensing’s model (Table 1) as an a priori coding framework. The six levels of the model were defined as themes, while the listed barriers and incentives were defined as sub-themes. Two researchers independently coded the data to identify the most basic segments that could be assessed in a meaningful way about factors influencing implementation. During frequent meetings between the researchers, the codes were compared and the text fragments were sorted into the six themes and their related sub-themes in Microsoft Word. However, some of the data extracts on influential factors related to specific implementation strategies did not fit in the given themes and sub-themes. The model therefore had to be adapted, and finally consisted of seven themes and 22 sub-themes.

**Table 1:** Barriers to and incentives for change at different levels of healthcare^a^

Level	Barriers/ incentives
Innovation	Advantages in practice, feasibility, credibility, accessibility, attractiveness
Individual professional	Awareness, knowledge, attitude, motivation to change, behavioural routines
Patient	Knowledge, skills, attitude, compliance
Social context	Opinion of colleagues, culture of the network, collaboration, leadership
Organizational context	Organisation of care processes, staff, capacities, resources, structures
Economic and political context	Financial arrangements, regulations, policies

^a^Grol and Wensing’s multilevel model [18]

### Ethical considerations and informed consent

The study was conducted in accordance with the ethical principles for medical research involving human subjects as stated in the World Medical Association’s Declaration of Helsinki [22]. Before each interview, participants were informed about the purpose of the study, that participation was voluntary and that they could withdraw at any time for any reason. It was explained that the data would be anonymized and that the participants could insist on having any of their statements deleted from the record. All participants who were asked confirmed their willingness to participate and consented to the interviews being audio recorded. When transcribing the interviews, all names of persons, services, cities and municipalities were exchanged with anonymized identifiers. The decision to either participate or withdraw from the study had no impact on the professionals’ work situation. According to Norwegian legislation, interview studies about non-sensitive topics that do not include patients or other vulnerable research subjects do not require approval from the regional ethical committee.

## Results

Table 2 gives an overview of the different improvement projects described by the participants. The barriers and facilitators that were extracted from the interviews were related to characteristics of: (1) the innovation; (2) the individual professional; (3) the patient; (4) the social context; (5) the organizational context; (6) the political and economic context and (7) the implementation strategy. The first six themes describe generic factors that were mentioned when implementing improvements in PC (Table 3). The final theme describes barriers and facilitators that were mentioned in connection with specific implementation strategies (Table 4).

**Table 2:** Overview of improvement projects discussed during interviews

Improvement project	Service
Assessment of social needs (social network map)	H-PCU LMC-PCU
Bereavement care	LMC-GU LMC-PCU
Campaigning for palliative care in other health care services	H-PCU LMC-PCU PCT
Campaigning for including palliative care in national health care system	H-PCU
Developing standard procedures in PC	LMC-PCU
Establishing a PCU	H-PCU LMC-PCU
Establishing a professional network for nurses in cancer & palliative care	PCT
Evaluating care after death of patient (evaluation form)	LMC-GU
Implementing a checklist for multidisciplinary meetings	H-PCU
Implementing a municipality standard for end-of-life care in NHs	LMC-GU NH LMC-PCU
Implementing guidelines and national policy in PC	H-PCU
Implementing the LCP or other care pathways	NH H-PCU LMC-PCU
Improving staffs’ PC expertise	H-PCU LMC-PCU NH
PC to new patient groups	H-PCU
Staff support through reflection groups	H-PCU LMC-GU LMC-PCU
Symptom assessment (ESAS, pain body map)	H-GU H-PCU LMC-PCU LMC-GU PCT

*GU* geriatric unit, *H* hospital, *LCP* Liverpool Care Pathway, *LMC* local medical center, *NH* nursing home, *PC* palliative care, *PCT* palliative care team, *PCU* palliative care unit

**Table 3:** General barriers to and facilitators for implementing changes in palliative care

Theme	Subtheme	Barriers	Facilitators
Innovation	Credibility	• Tool not validated	• Pilot test tool
	Advantages in practice	• Not apparent to staff	• Apparent to staff
	Accessibility	• No storing system for paper copies	• Paper copies available • Part of electronic documentation system
	Responsibility	• One person responsible	• Sharing responsibility
	Attractiveness	• Time consuming • At odds with care philosophy	• Simplicity of tool
Individual professional	Motivation to change	• Innovation not perceived as attractive • No regular training • Not involved in planning • Part-time/ temporary staff	• Innovation perceived as attractive • Regular training • Involved in planning
	Knowledge and expertise	• Lack of PC expertise	• PC expertise
	Confidence	• Lack of confidence	• Training in PC • Access to advice from experts
Patient	Lack of compliance	• Lack of motivation • Understate pain • High symptom burden • Reduced cognitive abilities	• Staff motivate patients
Social context	Leadership	• Distant management • Lack of leadership support • Nurses not represented in leadership group • Negative attitude to change	• Enthusiastic • Supportive • Knowledge of implementation and organizational change • Involve staff • Tailor change • Attentive • Anchor change in administration • Positive attitude to change
	Culture of change	• Lack of support from colleagues	• Openness
	Face-to-face contact	-	• Site visits and observation • Joint educational sessions
Organizational context	Resources	• Low staff/patient ratio • Lack of time	-
	Structures and facilities	• Lack of facilities • Changes in building structure	• Close proximity to collaborating services • Flexible admission system
	Expertise	• Lack of expertise • Lack of QI nurse	• Previous experiences with improvement projects • Low staff turnover • QI nurse: PC + educational skills
Economic and political context	Policy and legislation’s influence on the level of expertise in community health care services	• Lack of PC resource persons • Lack of educational training • “Adopted” staff	-
	Financial arrangements	• Lack of extra funding • The coordination reform	• Extra state funding • Hospital pays for medication • National activity-based funding system

*PC* palliative care, *QI* quality improvement

**Table 4:** Barriers to and facilitators for using specific implementation strategies

Subtheme	Code	Quotation
Implementation strategy: Educational strategies		
Timing	• B: Daytime	*We also have internal educational sessions every other week from 14.15 to 15.00. However, we see that it is difficult for the daytime staff to attend these sessions* (Assistant head nurse, H-GU).
	• B: Half-day (leaving the clinic)	Nurse H2: *Leaving* [the clinic] *towards the end of the day – that was the challenge with these half-day or two hour courses.* Nurse H1: *It is really frustrating for those who remain* [in the clinic], *who then have to take over the patients with all the responsibility this entails.* (Nurses, H-PCU)
	• B: After large changes have been implemented	*We were the ones that called out: “If these patients are supposed to be here from now on, then we need training”, and then the training was arranged a while after the fact. A nurse from that department came 2–3 mornings and gave us a light briefing* (…) *and it is very challenging to care for these patients* (Nurse 2, H-PCU).
	• F: Evenings	*We have arranged quite a few training courses in the evenings in order to reach everyone* (Head nurse, NH).
	• F: Half-day (easier to organize)	*In the beginning, we had full-day sessions. Since then, we have been arranging half-day sessions, because having them from noon to three-thirty is easier to organize* (Executive director, LMC-PCU).
	• F: Full-day	Nurse L3: *I think it was a good thing that we were taken out of the clinic, because then we didn’t have to feel stressed about going to the course.* Interviewer: *Being released for the whole day?* Nurse L3: *Yes, that worked much better.* (Nurse, LMC-PCU)
	• F: Arranged repeatedly	*We repeated the program a second time so that everyone would have the opportunity to participate despite working shifts* (QI nurse, LMC-GU).
	• F: Before (*large)* changes have been implemented	*In my experience, it is great to get good information and training in advance. That is something we see works well. Include them in whole day training sessions, so that we have plenty of time* (Head nurse, H-GU).
Funding	• B: Lack of funding	*We cannot afford whole days, i.e. we have only allowed ourselves two days* [of educational sessions]*. This is not much for a whole year* (Executive director, NH).
	• F: Hiring substitutes	*We finish this project formally this summer. The money has first and foremost been used to hire substitutes for the employees. When employees participate in educational courses, we can’t just empty out the ward. We need substitutes there during that time, because the patients are still there* (Executive director, LMC-PCU).
	• F: Extra project funding	
Organizational aspects	• B: High staff turnover	Physician (H-PCU): *We spent a lot of time on in-house educational sessions in the community services, but it just frittered away, so we gave it up. It was a waste.* Interviewer: Do you know why? Physician (H-PCU): *Yes, because they were replacing staff constantly. So you need to consider what you’re spending your resources on, and that was not the way to go.*
	• F: Mandatory attendance	*You must require it of them. It must be compulsory, like the fire drills, because these are compulsory, and we see that this works, because then they have to attend* (Head nurse, NH).
Implementation strategy: Local champions		
Personal characteristics	• F: Expert	*If there was someone who had the expertise, whom you could call and make an appointment with and say “today I need to learn this”, then you would learn it, but that you could also call them back on the phone if you needed to.* (…) *Because if you are implementing something new, then it is a good idea to have someone who knows it better than others* (Physician, H-PCU).
	• F: Attitude towards project	*I think it is very important to choose the right people. One thing is the knowledge aspect, but another important factor is believing in the project and having the guts to follow through with it.* (QI nurse, LMC-GU)
	• F: Legitimacy	*And that this person has legitimacy in the work environment and is available. This makes implementation a lot easier* (Physician, H-PCU)*.*
	• F: Availability	
Organizational aspects	• F: Regular updates	*The pain resource nurses are summoned regularly throughout the year by a nurse anesthetist* (…) *There they are updated on the latest information about assessment and treatment* (Head nurse, H-GU).
	• B: Lack of opportunity to disseminate knowledge	*I’ve learned things that I should share with my colleagues, but there hasn’t really been room for it during our regular training days* (Head nurse assistant, H-GU).
Implementation strategy: Formal meetings		
Timing	• B: Too few staff meetings	*It is a challenge to reach everyone. It is a challenge to reach staff working the night shift; I only have two meetings a year with them* (Executive director, NH).
Group size	• B: Large	*It was in the physicians group that we managed to create a shared environment. While the nurses…there are so many of them.* (…) *It was easier to arrange [meetings], because we were not that many physicians* (Physician, H-PCU).
	• F: Small	
Organizational aspects	• B: Varying attendance rates	Physician (PCT): *We experienced that some municipalities were unable to send physicians* [to regional meetings]*, but then the physicians would ask us to come extra and participate in their internal meetings and educational sessions and we tried to accommodate them.* Interviewer: *Sounds like you made a great effort to make this happen –* Physician (PCT): *Yes, but it was very time consuming.*
	• F: Arrange additional meetings	
Implementation strategy: Reminders		
Type of medium	• B: E-mail/ phone	*I’ve tried e-mailing and phoning, but there is something about getting to see the person who asks about these things face-to-face. I think that works better. I think there is something about the psychology in that* (Physician, H-PCU).
	• F: Face-to-face	
	• F: Laminated cards	*It should be a smooth pedagogical program in combination with, in my opinion, something written that is easily available, to help us remember, as we have with the* [name of] *project. Something that can lay there readily available. Something laminated* (Head nurse, H-GU).
Frequency	• F: Repetition	*And I had to go again and again* [to remind staff] (Physician, H-PCU).
Social context	• F: Personal relationship	Interviewer: *You said you knew them personally, do you think this had an effect?* Physician (H-PCU): *Yes, I think so. That I had a personal connection to them.*
Implementation strategy: Change of professional role		
Educational policy	• F: Improved general education	*The nurse competence is much greater now than before. They learn much more during general training, which makes it easier to give them more responsibility* (Physician, H-PCU).
Gradual implementation	• F: Gradual transfer	*Often, there will be a small transition, it* [the change in tasks] *is not implemented suddenly from one day to the next, but you observe how things go* (Physician, H-PCU).
	• F: Trial period	
Staff involvement and motivation	• F: Involving staff	*It is not just one person who decides anymore. Now it is more something you discuss in the group until you find a solution* (Physician, H-PCU).
	• F: Motivated staff	*They had to be willing to attend [educational] sessions, so we tried to find staff members who seemed motivated to take on this task* (Head nurse, LMC-GU).

*B* barrier, *F* facilitator, *GU* geriatric unit, *H* hospital, *LMC* local medical center, *NH* nursing home, *PCT* palliative care team, *PCU* palliative care unit

### Innovation

By *innovation* we mean “the object of the implementation process” [23]. The experiences of the services in implementing improvements in PC ranged from large innovations such as starting up a palliative care unit (PCU), to small innovations such as introducing a symptom assessment tool (Table 2). Under this theme we will consider influential factors related to the Edmonton Symptom Assessment System (ESAS), the Liverpool Care Pathway (LCP) and the change from hospice philosophy to palliative medicine in specialist PC. ESAS is the standard symptom assessment tool in PC in Norway [14], and three of the services had tried to implement this tool. ESAS has nine 11-point numerical rating scales (0–10) assessing the intensity of pain and other common symptoms in PC patients [24]. Two services had tried to implement the Liverpool Care Pathway (LCP), which is an integrated care pathway for the care of a dying patient, with several sections documenting observations, decisions and interventions [25]. Credibility, advantage, accessibility, sharing responsibility and attractiveness of the innovation were mentioned as barriers to and/or facilitators for implementing these innovations.

#### Credibility

One challenge to implementing PC tools in nursing homes and LMCs, was that few tools are specifically tailored to primary care. A head nurse at the LMC that specializes in dementia mentioned that *“the tools might be validated on patients in hospital, younger perhaps, so it is a challenge that our patients are affected by dementia”.* For instance, patients with dementia were often not able to fill in ESAS. The LMC-GU solved this by having staff pilot-test ESAS and then fill in the forms for the patients who were unable to do so themselves. During the pilot, each staff member filled in a form for the same patient and then they discussed the results. This helped ensure that staff had the same understanding of the numbers when they assigned scores, which enabled a level of consistency that increased the credibility of ESAS. The head nurse commented: “*Even though this form was not very suited for the frail nursing home patient, it became a very useful communication tool between health care providers”*.

#### Advantage

The two LMCs had experienced difficulties in implementing ESAS because not all staff members saw the advantage of using this tool. Staff was therefore not motivated to spend extra time getting the patients to fill in the form. However, their motivation increased when the physicians started asking for the patients’ ESAS scores. The nurses then realized that the data in the completed forms was used by the physicians.

#### Accessibility

Always having paper copies available was reported as a facilitator for implementing tools. However, the use of paper versions of tools was not always perceived as an advantage. One of the LMCs experienced that not having a good system for storing the completed LCP paper forms was a barrier to using this tool. Several participants therefore said that having the new tools integrated into the electronic system would be a facilitator. The information gathered by the tools would then be easily accessible to staff.

#### Sharing responsibility

Another barrier regarding the unsuccessful implementation of the LCP, was that only one nurse per patient was responsible for filling in the LCP form. A physician commented that she thought it would have been easier to implement the LCP if two nurses had been given the responsibility of filling in the form together. Sharing responsibility was therefore perceived as a facilitator for implementing the LCP.

#### Attractiveness

The innovation was not perceived as attractive if it was too time consuming. The simplicity of ESAS was therefore mentioned as a facilitator to using it, while the number of different sections of the LCP was pointed out as a barrier. The innovation was also not perceived as attractive if it was not in line with staff’s care philosophy. A nurse in the H-PCU says:

*When I started working at* [the PCU]*, the hospice philosophy – that is, our overall aims and values – were very clear. They influenced every context.* (…) *Now, this hospice philosophy, it’s gone. And we’re now over in a more acute palliative medicine mindset.* (…) *The focus is purely medical, getting the patient through the system at a much higher pace, so to speak* (Nurse 4, H-PCU)*.*

One of the physicians confirmed that the number of days a patient spends in the H-PCU has been cut in half. However, the nurses’ critical comments about the shift in specialist PC focus were intertwined with comments about an extensive downsizing and reorganization process in the cancer clinic the year before. According to the nurses, these changes had increased the work load at the PCU to such an extent that both their work environment and patient care had suffered. The nurses commented that they did not have sufficient time to provide necessary PC. Therefore, the nurses did not perceive the shift in PC focus as attractive in the given situation.

### Individual professional

Three aspects were mentioned regarding how individual professionals can influence a successful implementation: motivation, PC expertise and confidence.

#### Motivation

All interviewees emphasized that staff members’ motivation was an important factor for success, regardless of type of intervention. Several factors may have influenced their motivation. One factor mentioned was the perceived attractiveness of the innovation. A second was receiving regular training in PC. Third, whether or not staff members were involved in the planning of the implementation process was central to their motivation. Finally, the number of working hours per week could influence their motivation. The executive director of the nursing home found it difficult to involve temporary staff and those working part-time in the implementation of improvements. The difficulties concerned dissemination of information to these staff members and ensuring that they were loyal to the decisions that had been made. In her opinion, “*people in part-time and temporary positions don’t have the same sense of responsibility”.*

#### Palliative care expertise

Lack of knowledge about and professional skills in PC were mentioned by several interviewees as barriers to improving PC in the services. For instance, ESAS was not used as planned in one LMC because some staff members lacked knowledge about the tool. For the same reasons, an attempt to implement the LCP proved unsuccessful. Several of the interviewees stated that the staff lacked basic PC skills, such as the ability to assess when a patient is imminently dying:

*It is a bit difficult to realize when the patient has only a short time left. Suddenly the patient is dead, and you’re like “oh, I guess we didn’t do it this time either”* (Nurse, LMC-PCU).

According to one physician, an important facilitator for implementing the LCP would therefore be that the staff has sufficient competence and experience to assess the signs, and enough confidence to acknowledge that someone is dying.

#### Confidence

Several nurses expressed that they felt anxious about being responsible for terminally ill patients. Training in PC was reported as an important measure to improve the confidence of staff. Another valuable initiative was having access to advice from experts. At the nursing home and the LMC-PCU, staff could call the nursing home physician at any time, and this created a feeling of security for them.

### Patient

Patients’ influence on the success of an improvement project was only mentioned in relation to ESAS. Patient compliance is essential when implementing ESAS. Compliance may be influenced by the patients’ motivation, symptom burden and cognitive abilities. One nurse commented: *“Some patients will say “Oh no, not one of those forms again!””* The nurses often had to spend time motivating the patients, and this got low priority when the ward was busy. The patients’ lack of motivation for filling in assessment tools can therefore be a barrier to using these tools. Another problem was that sometimes older patients would understate the pain they experience, explaining to the nurses that *“some pain is to be expected when I’m this old”*. Patient compliance is also affected by their symptom burden and performance status. Finally, diminished cognitive abilities due to dementia or delirium may affect patient compliance and prevent the use of self-report tools.

### Social context

Barriers and facilitators that relate to the social context are connected to leadership, culture of change and face-to-face contact.

#### Leadership

The role of the management was mentioned in all interviews as crucial to the success of an improvement project. Participants mentioned several actions that leaders must take to facilitate such projects. First of all, the leader must be enthusiastic about the change and signal that the project is important for the service, for instance by allocating time for staff members to work on the project. A second necessary action is to plan the project and its introduction in the service well. In order to do this, the leader must have knowledge of and experience in implementation and organizational change. Also, he/she should talk with the staff in order to identify the areas with the greatest need for improvement. Tailoring the implementation process to the specific service and target group was also mentioned as an essential part of the planning process. Next, the leader should involve the staff, preferably at an early stage. One head nurse commented:

*The most important tool you have in any improvement project is the staff. If you don’t have them on your side, or they understand what is going on, then the whole thing is practically a waste of time* (Head nurse, LMC-GU).

One manager recommended a gradual approach, i.e. involving nurses higher up in the hierarchy first, while another manager recommended involving as many staff members as possible in the early phase. Furthermore, the leader needs to be present and attentive, i.e. keep a close eye on the process and ask for feedback from staff about how the project is going. He/she should also make a point to praise staff when their effort is contributing to implementing the improvement. Finally, the leader should ensure that the project is evaluated and then anchored in the administration.

Two barriers connected to leadership were described by nurses working in one PCU. The first was distant management that led to a lack of detailed understanding of the tasks involved in patient care. The consequence had been unclear directions about the changes that should be implemented and what was expected of staff. One nurse commented:

*We have been told that we need to do less of something. This we’ve been told many times. However, when we ask which tasks to reduce, we are told that we need to figure this out for ourselves. We who are in the clinic see that it is not possible to reduce the care of this patient group, in fact they are even more demanding now. So we are being subjected to conflicting demands* (Nurse, H-PCU).

The second barrier was that these nurses were not represented in the management group and were therefore not involved in the decision making processes regarding organizational changes. During a large reorganization process, the nurses experienced that they were not heard by the management when they tried to raise concerns about the process. According to the participants, this had resulted in frustration, resistance to the change and decreased work satisfaction amongst the nurses in this service.

According to a QI nurse, the leader’s perception of whether the change was good or bad also influenced how willing the staff was to accept and want the change. Thus the leader sets the tone for the culture of change that permeates the service.

#### Culture of change

Having a working environment that is characterized by trust and open communication was reported as a facilitator:

*You need an environment that is open for feedback. Things must not be swept under the carpet. The environment needs to be open, so that you in fact can disagree and be heard* (Chief executive, nursing home).

A barrier to a constructive culture of change was lack of support from colleagues. Not all staff members understood why some of their colleagues were given more training and responsibilities that took them away from daily clinical work. This lack of understanding from colleagues had been stressful for staff members involved in improvement processes.

#### Face-to-face contact

Several interviewees mentioned that if you want to improve PC in your service through collaboration with another service, it is an advantage for staff in the two services to meet face-to-face. This facilitates subsequent contact when necessary. Strategies to ensure this include site visits and observation and joint educational sessions.

### Organizational context

Resources, structures, facilities and expertise are keywords for barriers and facilitators connected to the organizational context.

#### Resources

Staff/patient ratio and how this affects the time available for working on improvement projects have significant influence on whether or not a project succeeds. One executive director and one physician mentioned the increased demand for documentation as a barrier. This meant that staff had more administrative work to do in addition to the clinical work. The clinical workload had also increased because the patients admitted to all the services were generally in poorer condition than before. Despite this, there had not been an increase in the staff/patient ratio. The time and motivation available for working on implementing changes were therefore limited.

#### Structures and facilities

Proximity to collaborating services was mentioned as a facilitator for starting up a PCU at one of the LMCs. Another structural facilitator was a very flexible admission system. Both the hospital and designated offices in the municipality may refer patients to this PCU.

Lack of appropriate facilities was reported as a barrier to following certain recommendations in the municipality standard for PC in nursing homes. According to the head nurse at one of the LMCs, some nursing homes did not have a private area for relatives to say goodbye to their deceased loved ones.

At the regional hospital, one of the physicians mentioned that changes in the building structure of the hospital had become a barrier to successfully campaigning for PC:

*…the hospital itself has changed from being one big building to several big buildings. We used to meet colleagues in the canteen. But now we’re too busy, so we never go to the canteen, and if you do, you go to different canteens, so you don’t meet colleagues like you used to. The lobbying you could do earlier, you can’t do that anymore* (Physician, H-PCU).

Thus, after the rebuilding, it had become more difficult to notify colleagues informally about the PC service.

#### Expertise

The QI nurse at the LMC-GU mentioned that their previous experience with improvement projects as well as low staff turn-over had been great advantages when initiating new improvement projects. Staff had built up expertise in implementing changes, and they had been able to avoid previous pitfalls.

Another facilitator was having a resource person with expertise in both PC and education. The LMCs and the nursing home all had QI nurses who were responsible for increasing the expertise of the staff through in-house training and discussions, in addition to supervising students. These nurses devoted their time to improvement projects and teaching and did not do clinical work. The nurses at the H-PCU said that losing their QI nurse during a reorganization process was a major barrier to improving their service.

### Economic and political context

Barriers and facilitators connected to the economic and political context concern the level of expertise in community health care services and financial arrangements.

#### Policy and legislation’s influence on the level of expertise in community health care services

The participants mentioned three barriers regarding how policy and legislation influence the expertise of staff. First, according to one manager (LMC-GU), there are few nursing homes in Norway who have QI nurses or other resource persons with PC training. She commented that there should be a resource person for PC in all nursing homes. In addition to being a financial issue, this also has to do with regulations on what sort of professionals should be available in primary care.

Second, there is also a lack of local champions with educational training in nursing homes. Availability of local champions would have made it easier to implement improvements when it is necessary to increase the expertise and competence amongst staff. Since nurses often have to teach colleagues, two of the managers mentioned that there should be more focus on educational skills in the nursing training. Then it would be easier to improve primary care services. This has implications for the content of the general training for nurses, which is partly regulated by the national government.

Finally, Norwegian legislation influences the ability of health care services to increase expertise. For example, the executive directors in community health care services cannot always decide whom to employ:

*I’ve inherited a lot of employees from two other services that were shut down when we moved in here. So it’s a long-term job to build up the expertise I think should be here. I can’t fire people; I’m not allowed to* (Executive director, LMC-PCU).

The LMC did hire some nurses with PC experience when establishing their PCU. However, the executive director was not able to hire as many experienced nurses as she would have liked to. Norwegian legislation requires employers to favor redundant staff if there is an opening for similar positions in the organization [26]. The consequence is that community services rarely can increase expertise quickly by hiring experienced staff, if there is redundant staff in the municipality with health care training.

#### Financial arrangements

Funding was mentioned in all interviews as an important factor for implementing improvements. The LMCs have limited budgets. Getting extra state funding was therefore a key facilitator for establishing the PCU. Part of this funding was used for staff training. A second financial facilitator was that the local hospital agreed to pay for the most expensive medication for the patients admitted to the LMC. However, other additional costs were not reimbursed. Consequently, the LMC could not establish the planned ambulatory PCT.

A national facilitator for stimulating the expansion of PC in hospitals has been to include PC in the national activity-based funding system. Other hospital wards get extra reimbursement if they refer patients to the specialist PC services in the hospital, giving them an economic incentive to do so. According to one physician, the PCU had seen an increase in the number of referred patients after this incentive was introduced.

Finally, the financial implications of the national Coordination Reform were mentioned as a barrier to improving PC in community services. After the reform was introduced in 2012, patients have been discharged from specialist services earlier than before. The idea is that specialist services should focus on acute specialist procedures only, while relevant follow-up care is to be provided by primary care services. However, not all municipalities have the capacity to receive patients in need of high level care. Municipalities, fully aware of their shortcomings, sometimes refuse to receive their patients after they have been declared ready to be discharged from specialist services. This results in a €475 fine per day per patient, paid by the municipalities to their regional health care authorities. According to both nurses and physicians at the H-PCU, this financial incentive had also led to some municipalities accepting the return of patients in order to save themselves this expense, in spite of their lack of expertise and resources. The end result had been negative for both the patients and the staff working in the hospital PCU:

*(…) we see that the patients come back right away.* (…) *with stories about how they have experienced others taking over the responsibility, with drastic consequences. These* [stories] *should actually have been recorded, because they say something about the lack of expertise and resources that patients meet, which makes them dread being discharged again, and it is hard for us to deal with, too* (Nurse 1, H-PCU).

The speed in which the patients were readmitted to the hospital had made the nurses call it the *“revolving door effect”*. Although the municipalities had accepted the patients back after the H-PCU had completed treatment, the primary care services were unable to provide the adequate care and the patients were soon sent back to the hospital. However, not all municipalities allowed this *“revolving door effect”*. The nurses at the H-PCU also described how some municipalities would pay the fine and let the patient stay in the hospital.

### Specific implementation strategies

Implementation strategies are “methods or techniques used to enhance the adoption, implementation, and sustainability of a clinical program or practice” [27]. The interviewees mentioned several specific influential factors that were connected to using particular implementation strategies, such as: educational strategies, local champions, formal meetings, reminders and changing professional roles.

#### Educational strategies

Examples of educational strategies used were lectures and workshops. The facilitators mentioned were connected with frequency, mandatory attendance, timing and funding, while the barriers were related to timing, funding and staff turnover.

According to the interviewees it was easier to get staff to participate in educational sessions if the training was repeated several times and if the leader made it clear that the training was mandatory. The latter was especially important in getting part time staff to participate.

Several interviewees highlighted the timing of the educational intervention. The nurses in both PCUs had all experienced that necessary training had been arranged *after* large changes had already been implemented. Stress due to lack of training could have been avoided if the training had started earlier. A second timing issue concerned the time of day the session was arranged. The head nurses of the nursing home and the H-GU mentioned that stepping out of the clinic during the daytime was difficult for the nurses, so evening sessions were better. The nurses of both PCUs also found it difficult to leave the clinic for short sessions, so they preferred full-day sessions to half-day sessions. On the other hand, management found half-day sessions easier to organize.

Another facilitator was hiring substitutes when permanent employees attended educational sessions. The PCU at the LMC was able to do this due to extra project funding from the government. This made it possible to arrange courses and seminars once a month during the first year. The nursing home, however, did not have extra funds for education. Their budget only allowed for two days of educational training a year. Lack of funding had therefore been a barrier to using educational strategies.

Another barrier was high staff turnover. One physician commented that, in her opinion, *in-house* PC training of staff in primary care services was basically a waste of time. This was because high staff turnover in the primary care services meant that they have a never-ending need for basic seminars in PC. Specialist services did not have the resources to meet this demand. Instead, they provided *regional* educational sessions once a year.

#### Local champions

One facilitator mentioned was the presence and characteristics of a local champion; a person with special training who can tutor colleagues. According to one physician (H-PCU), the local champion needs to be an expert in whatever is being implemented. He/she has to be available when staff needs help with a tool or a computer program. It is also important that this person has legitimacy in the department, i.e. that the staff knows the person and believes in his/her expertise. According to a QI nurse (LMC-GU), having local champions on each ward gives the staff ownership to a project. Another facilitator was having the local champions meet for regular updates. However, it could take a while before they had the opportunity to disseminate what they learned to their colleagues.

Choosing the right staff members as local champions was mentioned as important. However, this did not necessarily have to be those who were most interested in the project. The management in one service (LMC-GU) chose staff members who initially were negative towards a project. By motivating them to join the project, the management prevented these staff members from thwarting the progress of the project.

#### Formal meetings

Timing, group size and attendance rates were perceived as influential factors for using formal meetings as an implementation strategy.

All managers mentioned that they used previously scheduled staff meetings to disseminate information about changes to be implemented in the services. However, this is not always an efficient strategy for reaching all staff groups. For example, the executive director at the nursing home had only two meetings a year with the night shift staff. She commented that this made it difficult to inform this group about projects in due time.

The size of the professional groups working in the services was an issue for arranging formal meetings. Two PCUs had been able to increase their physicians’ knowledge of PC by arranging case review meetings between the physicians in the two services. It was possible to arrange these meetings because there were so few physicians in each service. On the other hand, arranging similar events among the nurses had been impossible because of their high number.

Varying attendance rates had been a challenge for a PCT arranging regional information meetings for health care professionals. In some municipalities, the PCT was sometimes invited by the GPs to do the session at their specific work place because their participation at designated regional meetings had been hindered. These extra sessions were perceived as very time consuming.

#### Reminders

Type of medium and frequency was mentioned as influential factors regarding reminders. The head nurse of the H-GU found that having laminated instruction cards in plain sight facilitated the implementation of new routines. One physician did not find e-mails or phone calls helpful in reminding services of new obligations. Making direct contact with staff was more effective. However, even reminding someone in person had to be done several times to ensure that staff remembered the new obligations.

#### Change of professional roles

Changing professional roles means modifying the role, tasks and responsibilities of professionals. For instance, increased patient loads had resulted in the need for other professions to do some of the physicians’ tasks. Facilitators mentioned were connected to educational policy, gradual implementation and staff involvement and motivation.

According to a physician and a manager, it had become easier to delegate tasks to nurses, because their general training is more extensive than it used to be. For more comprehensive duties, such as nurses taking over administration of chemotherapy, the physician recommended a gradual transfer of tasks and having a trial period.

One physician mentioned the importance of involving the relevant professionals. In her service, the professionals involved would first have a discussion about what could be done to solve an issue. By doing this, the professionals who took over tasks would take part in the decision to change their responsibilities and also in planning the execution of this change. For some tasks, a motivated staff was a necessary precondition for changing professionals’ responsibilities. This factor was especially important if the staff had to undertake additional training to increase their expertise regarding the new task.

## Discussion

In this article we have reported the findings of a qualitative study about barriers and facilitators that influenced the implementation of improvements in PC in six different health care services in Norway. The participants mentioned several factors that influenced specific implementation strategies, such as educational strategies, formal meetings, reminders and changing professional roles. However, most of the barriers and facilitators highlighted by the participants were not mentioned in connection with specific implementation strategies. These factors were related to the innovation, the individual professional, the patient, the social context, the organizational context and, finally, the economic and political context.

Staff involvement and support from the leader were emphasized as important facilitators in all interviews. When these factors are absent, staff’s motivation for participating in the change may suffer and they may resist the change, as has been found in other health care fields [28]. Staff shortage and lack of funding have also been identified as barriers by others [11, 29] and it is therefore no surprise that improvement projects in PC require extra financial and staff resources. This is especially true for strategies that require health care providers to step out of ordinary clinical work.

Even if most of the factors identified in our study are not unique to PC, the significance of some of the barriers and facilitators may differ from other fields of health care. For instance, lack of patient compliance is also mentioned as a barrier to implementing improvements in diabetes [30] and in prevention of bacterial endocarditis [31]. However, this barrier may be an even greater challenge in PC due to the poor physical and cognitive condition of the patients. This may require a different approach when implementing improvements that require patient participation.

Three barriers can be argued as being particular to PC. First of all, the diverse characteristics of patients in need of PC imply that not all symptom assessment tools developed in this field are suited for all patient groups. For instance, tools used to assess symptoms in young patients with cancer are not necessarily appropriate in a population of patients with dementia. Therefore, there may be a validity issue limiting the implementation of these tools.

The second barrier concerns the tension that occurs when changes are at odds with the holistic hospice care philosophy. The shift described by the nurses in this study towards a more medical focus in specialist PC, has happened throughout Europe, the US and Australia [32–35]. Several of the nurses who were critical of the direction that PC in their service was taking, had many years of experience from the field. This means that they would have been socialized in the hospice ideology characterizing the service when they started working there. Their long track record in PC may therefore have made it more difficult to embrace the emphasis on acute palliative medicine and medical treatment. This shift in specialist PC has been developing in Norway over the past 15 years, and Strømskag [36] describes how it has been debated by different professional groups. Most advocates for the shift have been found in the medical profession, while nurses have been more critical. However, the nurses participating in the present study were not negative towards all changes the shift implied. For instance, they agreed with the aim that new patient groups should have access to PC. The problem seemed to be the way this change had been implemented, for example that they had to ask for training to deal with the challenges the new patient groups represented. The nurses’ comments on the shift in PC were also intertwined with comments about the Collaboration Reform and the recent downsizing and reorganization process in their clinic. The tension they expressed must therefore also be seen in the light of other national and local changes being initiated simultaneously. The nurses felt that their opinions were not considered as they were not represented in the management group planning how to deal with these changes. They might have been more positive towards the shift in PC if they had been involved in the planning of specific changes.

Finally, lack of PC expertise may hinder implementation of improvements. Many innovations in this field, e.g. the Liverpool Care Pathway, require PC skills and experience. In Norway, physicians can specialize in PC through the Nordic Specialist Course in Palliative Medicine [37], while there is no formal specialist training in PC for nurses. However, there are continuing educational courses for nurses in PC, and some specializations are relevant, such as oncology and geriatrics [14]. Despite this, only 39 % of nurses working in PCUs in nursing homes have any form of postgraduate education [14], and 26.1 % of staff in nursing homes and home care are unskilled labor [38].

The lack of PC training of staff working in primary care was one of the reasons why the interviewees working in specialist PC were critical of the changes initiated by the Coordination Reform [39]. The reform was not only aimed at improving PC, and health care providers in other disciplines in Norway, such as stroke [40], psychiatry [41] and multiple sclerosis [42], have claimed that insufficient training of health care providers in primary care is a problem. Since the interviews were conducted, projects to improve general PC expertise in community care have been developed, such as the establishment of cancer coordinators in many municipalities and networks of professionals coordinated by the four regional centers for excellence in PC [14].

The Liverpool Care Pathway is one innovation that has been the topic of educational interventions directed at health care providers in Norway during the past few years. The LCP was introduced in Norway in 2006, and is now being used in 337 different institutions and services [14]. Two of the services were trying to introduce the LCP when the interviews for the present study were conducted in 2012. At that point, they had not been successful, and they pointed out several barriers connected to characteristics of this innovation that had hindered the implementation. This was before the critical media coverage of the LCP in the UK. There has not been any negative media attention on the care pathway in Norway, and implementation has been a success in many services. Even so, the two services in this study decided to give up this project in the end. When asked about this during an interview for the third phase of the IMPACT project, a representative from one of the services stated that the media coverage in UK was one of the main reasons. Based on the UK experience, the Directorate of Health has proposed a project to evaluate the implementation and use of the LCP in Norway [14]. A revised document even more closely adapted to the Norwegian context will be launched [43].

### Strengths and limitations

A strength of this study is that it was conducted in a variety of settings that provide PC; a GU and a PCU in a regional hospital, a PCT in a local hospital, LMCs specializing in PC and dementia, and a nursing home. This means that the findings may be relevant to a variety of services addressing patients with cancer and dementia. Furthermore, the interviews and focus groups revealed a wide range of different factors influencing implementation of improvements.

The insider-outsider question needs to be considered as a potential limitation when doing qualitative research [44]. The first author and the research assistant did not have any clinical experience in PC, and could thus be considered ‘outsiders’. As far as we could tell, this did not restrict the data collection. The participants in the focus groups seemed comfortable with the discussion format and all interviewees seemed to be willing and able to describe their experiences and express opinions on the topic. This was perhaps because both the first author and the research assistant knew some of the participants through their jobs at the European Palliative Care Research Centre and the Regional Center for Excellence in Palliative Care. On the other hand, the interviews conducted with people that we did not know, also provided rich data on the topic. After the interviews, several of the participants expressed that the interview had set in motion a process of reflection that might help them in later improvement processes.

The use of a retrospective interview method may represent a recall bias, with faulty memory and selective recall [45]. The interviewees may not have remembered all relevant barriers and facilitators. A second limitation is the small number of participating services, although this was similar to the other countries participating in the large IMPACT study. The relatively small geographical distribution of participating centers in only one region of Norway may also be viewed as a limitation. We do not know if saturation was reached; more barriers and facilitators might have been identified if we had interviewed health care providers in other services and in other regions of the country. On the other hand, Norway is a homogeneous society, both politically and socially, with almost all health care services funded by the government. Therefore, there is little reason to assume that any regional differences will make the findings from this study irrelevant for services in other parts of the country. The fact that many similar aspects were mentioned by many interviewees across services in the Central region of Norway also makes us believe that important barriers and facilitators were identified.

Another limitation of our study is that we lack data from home care services. In Norway, 13 % of all cancer deaths occur at home [46]. PC policy aims to increase this number [14], so home care services have a central role in PC and should have been included in the study. However, home care services have been included in the next phase of the IMPACT project. Data on barriers to and facilitators for implementing improvements in such services will therefore be presented in a later publication.

The gender imbalance among interviewees, with 19 of 20 being females, could be viewed as a limitation in the present study. However, in Norway overall, more than 70 % of leaders in health care and 84 % of health care personnel are women [47, 48] and maybe even more so in PC services. It was therefore appropriate that most of the participants we were able to recruit were female.

Finally, this analysis relies heavily on Grol and Wensing’s model of “barriers to and incentives for change at different levels of healthcare” [49]. According to Dierckx de Casterlé, “using a preconceived framework runs the risk of prematurely excluding alternative ways of organizing the data that may be more illuminating” [50]. However, Grol and Wensing’s model was used in the second round of data analysis, after the data had first been analyzed inductively. The results from the second round were compared with the findings in the report from the first round of data analysis, to ensure that no barriers and facilitators had been missed. Grol and Wensing’s model was also adapted in this study to capture the barriers and facilitators mentioned in connection with specific implementation strategies.

### Implications for clinical practice

Various models for translating results of research into clinical routines describe the analysis of barriers and facilitators as one of the first steps in the implementation process [9, 51, 52]. The results from this retrospective study have been used to tailor improvement strategies for services participating in the IMPACT intervention study [10]. The barriers and facilitators identified in this study also offer guidance to services not participating in the intervention study. Health care professionals should consider these factors when tailoring their own strategies for improving PC provision in their services. Even so, services may differ concerning which factors are most influential. There may also be factors not mentioned here that should be taken into consideration. Therefore, the results of this study could also encourage professionals to perform their own analysis of barriers and facilitators before engaging in an improvement project.

## Conclusion

This study shows that there is a wide range of barriers and facilitators that have to be considered when planning an improvement project in PC. When implementing a new assessment tool, an important consideration is whether or not the tool has been validated for the relevant patient group, and to what degree patients need to be involved when using the tool. Leaders should involve staff early in the improvement process, ensure that they have the necessary training in PC and that the change is consistent with the staff’s philosophy of care. Some barriers and facilitators are related to specific implementation strategies. We therefore advise services to pay particular attention to the factors associated with the strategy they have chosen to use.

## Abbreviations

B, barrier; F, facilitator; GU, geriatric unit; H, hospital; LCP, Liverpool Care Pathway; LMC, local medical center; NH, nursing home; PC, palliative care; PCT, palliative care team; PCU, palliative care unit; QI, quality improvement

## Additional file

Additional file 1: Interview guide. (DOCX 16 kb)

## References

[1] Radbruch L, Payne S (2009). White paper on standards and norms for hospice and palliative care in Europe: part 1. Eur J Palliat Care.

[2] Definition of palliative care. European Association for palliative care (EAPC). 2013. http://www.eapcnet.eu/Corporate/AbouttheEAPC/Definitionandaims.aspx. Accessed 23 Sep 2014.

[3] van der Steen JT, Radbruch L, Hertogh CM, de Boer ME, Hughes JC, Larkin P, Francke AL, Junger S, Gove D, Firth P, Koopmans RT, Volicer L (2014). White paper defining optimal palliative care in older people with dementia: a Delphi study and recommendations from the European Association for Palliative Care. Palliat Med.

[4] World Health Organization (WHO) (2003). Global action against cancer. Responding to the challenge of cancer in Europe.

[5] Edmonds P, Karlsen S, Khan S, Addington-Hall J (2001). A comparison of the palliative care needs of patients dying from chronic respiratory diseases and lung cancer. Palliat Med.

[6] Prince M, Bryce R, Albanese E, Wimo A, Ribeiro W, Ferri CP (2013). The global prevalence of dementia: a systematic review and metaanalysis. Alzheimers Dement.

[7] Stjernsward J, Foley KM, Ferris FD (2007). The public health strategy for palliative care. J Pain Symptom Manage.

[8] Bero LA, Grilli R, Grimshaw JM, Harvey E, Oxman AD, Thomson MA (1998). Closing the gap between research and practice: an overview of systematic reviews of interventions to promote implementation of research findings. BMJ.

[9] Grol R, Wensing M, Eccles M (2005). Improving patient care: the implementation of change in clinical practice.

[10] IMPACT - IMplementation of quality indicators in PAlliative Care sTudy. 2014. http://impactpalliativecare-assessment.eu/en/info. Accessed 30 Jan 2014.

[11] Davies N, Maio L, van Riet Paap J, Mariani E, Jaspers B, Sommerbakk R, Grammatico D, Manthorpe J, Ahmedzai S, Vernooij-Dassen M, Iliffe S. Quality palliative care for cancer and dementia in five European countries: some common challenges. Aging Ment Health. 2014;18(4):400–10. 10.1080/13607863.2013.843157 PMC3979441 24131061

[12] van Riet Paap J, Vernooij-Dassen M, Brouwer F, Meiland F, Iliffe S, Davies N, Leppert W, Jaspers B, Mariani E, Sommerbakk R, Vissers K, Engels Y (2014). Improving the organization of palliative care: identification of barriers and incentives in five European countries. Implement Sci.

[13] Norwegian Directorate of Health (2007). Nasjonalt handlingsprogram med retningslinjer for palliasjon i kreftomsorgen. [National action program with guidelines for palliative care for patients with cancer].

[14] Norwegian Directorate of Health (2015). Rapport om tilbudet til personer med behov for lindrende behandling og omsorg mot livets slutt. [Report on service provided to people in need of palliative care and end-of-life care].

[15] Andre B, Ringdal GI, Loge JH, Rannestad T, Kaasa S (2009). Implementation of computerized technology in a palliative care unit. Palliat Support Care.

[16] Fyllingen EH, Oldervoll LM, Loge JH, Hjermstad MJ, Haugen DF, Sigurdardottir KR, Paulsen O, Kaasa S (2009). Computer-based assessment of symptoms and mobility in palliative care: feasibility and challenges. J Pain Symptom Manage.

[17] Andre B, Sjovold E, Rannestad T, Holmemo M, Ringdal GI (2013). Work culture among healthcare personnel in a palliative medicine unit. Palliat Support Care.

[18] Andre B, Ringdal GI, Loge JH, Rannestad T, Kaasa S (2008). The importance of key personnel and active management for successful implementation of computer-based technology in palliative care: results from a qualitative study. Comput Inform Nurs.

[19] Malterud K (2011). Kvalitative metoder i medisinsk forskning: en innføring [Qualitative methods in medical research: an introduction].

[20] Braun V, Clarke V (2006). Using thematic analysis in psychology. Qualitative Research in Psychology.

[21] Atlas.ti Qualitative Data Analysis. http://www.atlasti.com/index.html. Accessed 15 Feb 2014.

[22] World Medical Association (2013). World Medical Association Declaration of Helsinki: ethical principles for medical research involving human subjects. JAMA.

[23] Demiris G, Oliver DP, Capurro D, Wittenberg-Lyles E (2014). Implementation science: Implications for intervention research in hospice and palliative care. Gerontologist.

[24] Bruera E, Kuehn N, Miller MJ, Selmser P, Macmillan K (1991). The Edmonton Symptom Assessment System (ESAS): a simple method for the assessment of palliative care patients. J Palliat Care.

[25] MCPCIL (2012). Liverpool Care Pathway for the Dying Patient (LCP). Supporting care in the last hours of life.

[26] Norway (2015). Lov om arbeidsmiljø, arbeidstid og stillingsvern mv. (Arbeidsmiljøloven) [The working environment act].

[27] Proctor EK, Powell BJ, McMillen JC (2013). Implementation strategies: recommendations for specifying and reporting. Implement Sci.

[28] Yevchak AM, Fick DM, McDowell J, Monroe T, May K, Grove L, Kolanowski AM, Waller JL, Inouye SK (2014). Barriers and facilitators to implementing delirium rounds in a clinical trial across three diverse hospital settings. Clin Nurs Res.

[29] Lynch T, Clark D, Centeno C, Rocafort J, de Lima L, Filbet M, Hegedus K, Belle O, Giordano A, Guillen F, Wright M (2010). Barriers to the development of palliative care in Western Europe. Palliat Med.

[30] Lawler FH, Viviani N (1997). Patient and physician perspectives regarding treatment of diabetes: compliance with practice guidelines. J Fam Pract.

[31] van der Meer JT, van Wijk W, Thompson J, Valkenburg HA, Michel MF (1992). Awareness of need and actual use of prophylaxis: lack of patient compliance in the prevention of bacterial endocarditis. J Antimicrob Chemother.

[32] Clark D (2000). Palliative care history: a ritual process?. Eur J Palliat Care.

[33] Bradshaw A (1996). The spiritual dimension of hospice: the secularization of an ideal. Soc Sci Med.

[34] James N, Field D (1992). The routinization of hospice: charisma and bureaucratization. Soc Sci Med.

[35] McNamara B, Waddell C, Colvin M (1994). The institutionalization of the good death. Soc Sci Med.

[36] Strømskag KE (2012). Og nå skal jeg dø: Hospicebevegelsen og palliasjonens historie i Norge [The history of the hospice movement and of palliative care in Norway].

[37] Nordic Specialist Course in Palliative Medicine. http://nscpm.org/. Accessed 16 Apr 2015.

[38] Norwegian Ministry of Health and Care Services (HOD) (2013). Morgendagens omsorg [Future Care].

[39] Norwegian Ministry of Health and Care Services (HOD) (2009). Samhandlingsreformen: Rett behandling - på rett sted - til rett tid [The Coordination Reform: Proper treatment – at the right place and right time].

[40] Øvergård RA. Hjerneslag rammer hele familien [Stroke affects the whole family]. In: Forskning.no. 2013. http://forskning.no/helsetjeneste-helseadministrasjon-hjernen-sykdommer-samliv-sosiale-relasjoner/2013/01/hjerneslag. Accessed 15 Apr 2015.

[41] Tønseth S. Samhandlingsreformen svekker psykisk helsearbeid [The Coordination Reform impairs mental health care]. In: Forskning.no. 2013. http://forskning.no/forebyggende-helse-helsepolitikk/2013/01/samhandlingsreformen-svekker-psykisk-helsearbeid. Accessed 15 Apr 2015.

[42] Enstad M. Dårligere rehabiliteringstilbud til personer med MS [Worse rehabilitation programs to people with MS]. In: Forskning.no. 2012. http://forskning.no/meninger/kronikk/2012/08/darligere-rehabiliteringstilbud-til-personer-med-ms. Accessed 15 Apr 2015.

[43] Oppdatering om LCP, vinter 2015 [Update on the LCP, winter 2015]. Regional Centre of Excellence for Palliative Care, Western Norway Regional Health Authority. 2015. http://www.helse-bergen.no/no/OmOss/Avdelinger/klb/Sider/Oppdatering-om-LCP,-vinter-2015.aspx. Accessed 15 Apr 2015.

[44] Corbin Dwyer S, Buckle JL (2009). The space between: on being and insider-outsider in qualitative research. IJQM.

[45] Vaus D (2006). RETROSPECTIVE STUDY. The SAGE Dictionary of Social Research Methods. SAGE Publications, Ltd.

[46] Cohen J, Houttekier D, Onwuteaka-Philipsen B, Miccinesi G, Addington-Hall J, Kaasa S, Bilsen J, Deliens L (2010). Which patients with cancer die at home? A study of six European countries using death certificate data. J Clin Oncol.

[47] Statistics Norway (2013). Health care personell, 2013, 4th quarter.

[48] Statistics Norway. Mot maktens tinder? [Towards the power peak?]. 2010. http://www.google.no/url?sa=t&rct=j&q=&esrc=s&frm=1&source=web&cd=1&ved=0CB0QFjAA&url=http%3A%2F%2Fwww.ssb.no%2Fbefolkning%2Fartikler-og-publikasjoner%2F_attachment%2F39406%3F_ts%3D132af1db018&ei=mXwvVbmmHMmKsAGmp4H4DQ&usg=AFQjCNF80DAA3lGTKjDkDMndZnND8VlfJg. Accessed 16 Apr 2015.

[49] Grol R, Wensing M (2004). What drives change? Barriers to and incentives for achieving evidence-based practice. Med J Aust.

[50] Dierckx de Casterle B, Gastmans C, Bryon E, Denier Y (2012). QUAGOL: a guide for qualitative data analysis. Int J Nurs Stud.

[51] Campbell NC, Murray E, Darbyshire J, Emery J, Farmer A, Griffiths F, Guthrie B, Lester H, Wilson P, Kinmonth AL (2007). Designing and evaluating complex interventions to improve health care. BMJ.

[52] Rogers EM. Diffusion of Innovation, 5 edn. New York Free Press; 2003

