# Supplementary material for: Barriers to and facilitators for implementing quality improvements in palliative care – results from a qualitative interview study in Norway
Source: BMC Palliat Care. 2016 Jul 15;15:61. doi: 10.1186/s12904-016-0132-5 (PMC4947264; doi:10.1186/s12904-016-0132-5)
Supplement: Additional file 1: — Interview guide. (DOCX 16 kb) [file 12904_2016_132_MOESM1_ESM.docx]

**Aditional file 1: Interview guide**

| **Theme** | **Aim** | **Questions / probes** |
| --- | --- | --- |
| 1. Identifying known improvement strategies | To identify strategies that have been recently used to improve (the organization of) palliative care in the particular setting. | Tell me about the organization you are in.  Probes:   - How does the organization differ from the organization in the past? - Can you give an example... - What was changed - How was it changed |
| 1. Identifying barriers and facilitators | To identify factors (barriers and facilitators) that influence strategies to improve palliative care in the setting. | What are barriers/is helpful to improvement strategies used in your setting to improve palliative care?  Probes:   - Can you tell more about... - How does that influence daily work? - Can you give examples... - What is your experience with... - Are there any other influencing factors you can think of? |
| 1. Identifying potential strategies | To identify potential (future) strategies to improve the organization of palliative care in the setting, taking the factors that influence the provision of palliative care into account. | So what are you going/would you like to do next in your service?  Probes:   - Why this strategy? - Can you think of the consequences of such a strategy being used? - What do you need to improve palliative care? - Would [example strategies] work in your setting? If so, why?; If not, why? |
| 1. Supplement | To identify important aspects in the provision of palliative care that have not been discussed yet. | What do you think is really good?  If you could recommend to other settings or countries a strategy to improve palliative care that works well in your setting, what would that be?  If you could think of existing strategies to improve palliative care that you wouldn’t recommend to other settings or countries, what would that be?  Is there anything else you would like to discuss? |
